# Unraveling the Role of BAG3 in Hepatic Fibrosis: Genetic and Biomarker Insights in Metabolic Dysfunction-Associated Steatotic Liver Disease (MASLD)

**DOI:** 10.3390/ijms262311286

**Published:** 2025-11-22

**Authors:** Benedetta Maria Motta, Alessandra Rosati, Mariano Festa, Anna Basile, Tommaso Sarcina, Maria Caterina Turco, Marcello Persico

**Affiliations:** 1 Department of Medicine, Surgery and Dentistry, “Scuola Medica Salernitana”, University of Salerno, 84081 Baronissi, Italy; bmotta@unisa.it (B.M.M.); arosati@unisa.it (A.R.);; 2 FIBROSYS s.r.l. Academic Spin-off, University of Salerno, 84081 Baronissi, Italy

**Keywords:** metabolic dysfunction-associated steatotic liver disease (MASLD), liver fibrosis, BAG3, *PNPLA3*, *TM6SF2*, non-invasive biomarker

## Abstract

Metabolic dysfunction-associated steatotic liver disease (MASLD) represents a major global health burden, with hepatic fibrosis being the strongest determinant of clinical outcomes. While genetic variants such as *PNPLA3* and *TM6SF2* are recognized drivers of disease progression, the downstream molecular mediators linking genetic susceptibility to fibrogenesis remain unclear. This study investigated the role of the stress-response co-chaperone BAG3 as a circulating biomarker of hepatic fibrosis and its association with *PNPLA3* and *TM6SF2* genotypes. In a well-characterized Southern European cohort of 146 MASLD patients, liver fibrosis was assessed using the FIB-4 index, serum BAG3 levels were quantified by ELISA, and genotyping for *PNPLA3* and *TM6SF2* variants was performed. BAG3 concentrations were significantly higher in patients with advanced disease and correlated positively with FIB-4 values. Carriers of the *TM6SF2* risk allele exhibited increased BAG3 levels, and a cumulative increase was observed with multiple *PNPLA3* and *TM6SF2* risk alleles. These findings suggest that BAG3 may represent a potential, non-invasive biomarker reflecting fibrogenic burden and a mechanistic link between genetic risk and hepatic fibrosis in MASLD.

## 1. Introduction

Metabolic dysfunction-associated steatotic liver disease (MASLD) has emerged as the most common cause of chronic liver disease worldwide, affecting approximately 25% of the global population [1]. It is characterized by excessive hepatic fat accumulation in the presence of cardiometabolic risk factors—such as obesity, dyslipidemia, insulin resistance, and hypertension—and may progress to hepatic inflammation, fibrosis, cirrhosis, and hepatocellular carcinoma (HCC) [2]. Among these, hepatic fibrosis is the strongest predictor of liver-related morbidity and mortality in MASLD [3].

In addition, the socioeconomic impact of MASLD and its related cardiometabolic comorbidities represents a growing global challenge in terms of healthcare expenditures [4].

Early diagnosis of liver fibrosis and appropriate clinical management are essential to prevent progression to cirrhosis and its complications and may justify screening in populations with cardiometabolic risk factors. The 2024 EASL–EASD–EASO guidelines advocate a stepwise diagnostic approach in at-risk individuals. Initially, a validated, non-proprietary blood-based score—such as the Fibrosis-4 index (FIB-4)—should be employed [5]. Subsequently, if fibrosis is suspected or patients belong to high-risk categories, non-invasive imaging techniques such as transient elastography are recommended to further assess fibrosis severity.

Although liver biopsy is no longer required for the diagnosis of MASLD, it remains essential for grading hepatic inflammation and excluding alternative causes of liver disease. Consequently, a major future challenge lies in the development of non-invasive, blood-based diagnostic tools capable of simultaneously identifying liver fibrosis and quantifying hepatic inflammation. Polygenic risk factors play a crucial role in MASLD, influencing both disease susceptibility and progression. Polygenic risk score (PRS) models, incorporating multiple genetic variants, enhance the prediction of MASLD risk and offer new avenues for precision medicine [6,7,8]. In this context, several SNPs have been robustly associated with MASLD.

The *PNPLA3* gene encodes a lipase involved in triglyceride hydrolysis in hepatocytes. A single nucleotide polymorphism (SNP), resulting in an isoleucine-to-methionine substitution at residue 148 (I148M), impairs PNPLA3’s lipolytic activity, leading to hepatic triglyceride accumulation, lipotoxicity, and fibrogenesis [9,10]. This variant is strongly associated with increased susceptibility to steatosis, steatohepatitis, advanced fibrosis, and HCC across diverse populations [11]. Importantly, PNPLA3’s pathogenic role extends beyond steatosis, influencing stellate cell activation and fibrotic responses [12].

Similarly, *TM6SF2* encodes a transmembrane protein that regulates hepatic very-low-density lipoprotein (VLDL) secretion. The E167K variant impairs lipid export from hepatocytes, promoting intracellular lipid accumulation [13,14]. While this defect paradoxically reduces circulating lipids, it exacerbates hepatic steatosis and fibrosis, thus contributing to progressive liver disease [15]. Both *PNPLA3* and *TM6SF2* variants highlight the critical role of hepatocellular lipid dysregulation in the pathogenesis of MASLD. Beyond lipid metabolism, recent attention has focused on cellular stress response proteins in the progression of liver disease.

Bcl-2-associated athanogene 3 (BAG3) is a stress-inducible co-chaperone that modulates proteostasis, autophagy, apoptosis, and cytoskeletal integrity by interacting with heat shock protein 70 (Hsp70) [16]. In physiological conditions, BAG3 supports cell survival under mechanical or metabolic stress by facilitating the clearance of damaged proteins and organelles [17]. In pathological contexts, aberrant BAG3 expression has been linked to fibrosis in tumors and cardiac tissues through modulation of fibroblast and macrophages activation, apoptosis resistance, and extracellular matrix remodeling [18,19,20,21,22].

Although the role of BAG3 in hepatic physiology is less well characterized, emerging evidence suggests that it may influence hepatocyte survival, autophagic flux, and hepatic stellate cell (HSC) activation—all critical processes in liver fibrosis development [23,24]. Stress conditions typical of MASLD, such as lipid accumulation, oxidative stress, and inflammation, may upregulate BAG3 expression, thereby impacting the fibrogenic microenvironment.

In particular, its induction in response to ER stress and unfolded protein response supports a model in which BAG3 functions both as a marker of hepatocellular stress and a participant in maladaptive fibrogenic signaling [25]. The chronic activation of such stress response programs may facilitate HSC activation and resistance to apoptosis, promoting extracellular matrix deposition and progression of fibrosis [23].

Given that *PNPLA3* and *TM6SF2* promote lipid retention and cellular stress in hepatocytes, BAG3 may represent a downstream effector that connects metabolic and genetic insults to fibrosis development. The possible interaction between *PNPLA3*, *TM6SF2*, and BAG3 pathways in the liver represents a promising yet still underexplored area of research. Both *PNPLA3* and *TM6SF2* variants contribute to hepatic lipid accumulation, promoting a lipotoxic environment characterized by endoplasmic reticulum (ER) stress, mitochondrial dysfunction, and reactive oxygen species (ROS) production [26,27,28]. These cellular stressors may upregulate BAG3 in hepatocytes in response to lipotoxic stress. BAG3 might play a dual role, initially preserving hepatocyte viability, but potentially contributing to fibrosis by promoting survival of activated HSCs and increasing extracellular matrix deposition [19,29].

Moreover, the dysregulated lipid handling caused by *PNPLA3* and *TM6SF2* mutations might potentiate BAG3’s stress-response pathways, creating a vicious cycle where impaired lipid clearance, hepatocellular injury, and fibrotic signaling reinforce one another. Understanding how BAG3 expression and function intersect with genetic determinants of MASLD could thus uncover additional biomarkers and therapeutic targets useful in improving disease stratification and intervention.

In this study, we explore the significance of BAG3 detection in patients’ sera in hepatic fibrosis within the context of MASLD and its interaction with key genetic variants *PNPLA3* and *TM6SF2*, aiming to unravel potential new insights into the molecular mechanisms driving disease progression.

## 2. Results

### 2.1. Clinical and Biochemical Characteristics

The study cohort included 146 patients with MASLD, of whom 121 (82.9%) presented without advanced complications, 23 (15.8%) had cirrhosis, and 2 (1.4%) were diagnosed with MASLD-related HCC. Given the very small sample size and the biological continuum between cirrhosis and HCC in MASLD progression, they were grouped together to highlight the trend toward advanced disease. The mean age was 62.9 ± 14.0 years, and 56.2% were male. As expected, patients with cirrhosis or HCC were significantly older and exhibited higher AST levels and FIB-4 scores, along with increased liver stiffness (LS) values. These patients presented elevated serum BAG3 concentrations, compared to those with uncomplicated MASLD (Table 1).

A complete version of the clinical and biochemical characteristics for the MASLD, cirrhosis, and HCC subgroups is available in Supplementary Table S1.

Indeed, notably, serum BAG3 levels were more than two-fold higher in the cirrhosis/HCC subgroup (143.8 ± 245.1 pg/mL) than in the uncomplicated MASLD group (61.8 ± 220.9 pg/mL; *p* = 0.011), indicating a potential link between BAG3 concentrations in the sera and disease severity.

### 2.2. Association Between Serum BAG3 and Fibrosis

**Figure 1:**
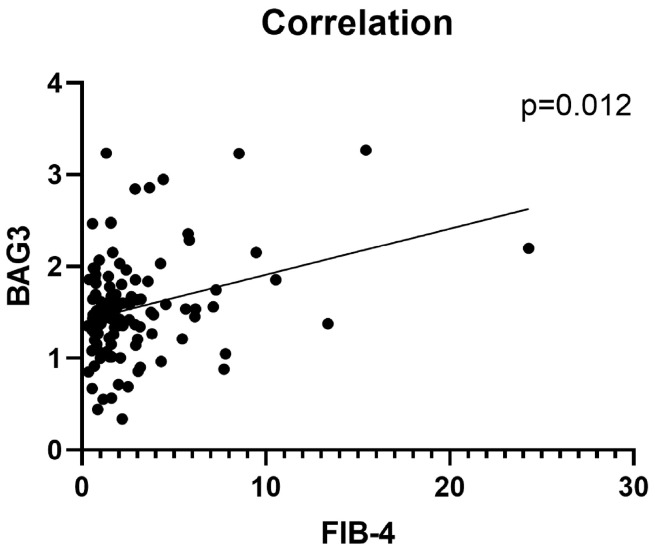
Correlation between serum BAG3 levels and liver fibrosis severity. The scatter plot shows the correlation between log serum BAG3 concentrations and FIB-4 index values in 146 patients with MASLD (Spearman’s ρ = 0.158, *p* = 0.012).

We observed a significant positive correlation between serum BAG3 and FIB-4 scores (Spearman’s ρ = 0.158, *p* = 0.012, Figure 1), suggesting that higher BAG3 concentrations are associated with advanced liver fibrosis.

**Figure 2:**
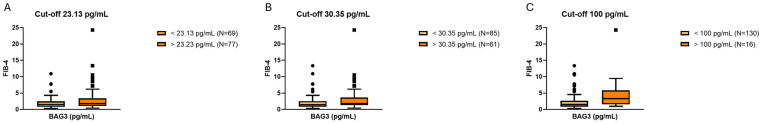
Stepwise increase in FIB-4 scores across stratified BAG3 serum concentration thresholds. Boxplots illustrate FIB-4 index values across increasing serum BAG3 concentration thresholds and categories, benchmarked against reference values from healthy controls. (**A**) BAG3 level cut-off of 23.13 pg/mL (mean in healthy controls, *p* = 0.023). (**B**) BAG3 concentration cut-off of 30.35 pg/mL (95th percentile in healthy controls, *p* = 0.008). (**C**) BAG3 concentration cut-off of 100 pg/mL (*p* = 0.004).

Stratified analyses confirmed this relationship across multiple BAG3 thresholds: individuals with levels above 23.13 pg/mL (mean level observed in healthy controls) had significantly higher FIB-4 values (*p* = 0.023, Figure 2A), with this effect becoming more pronounced at levels above 30.35 pg/mL (95th percentile, *p* = 0.008, Figure 2B) and 100 pg/mL (an arbitrary cut- off chosen among the highest values observed in healthy subjects, *p* = 0.004, Figure 2C), indicating a dose–response relationship between BAG3 expression and fibrosis severity. These thresholds were derived from previously published healthy control distribution [30] and are proposed as exploratory reference ranges for future validation studies.

**Figure 3:**
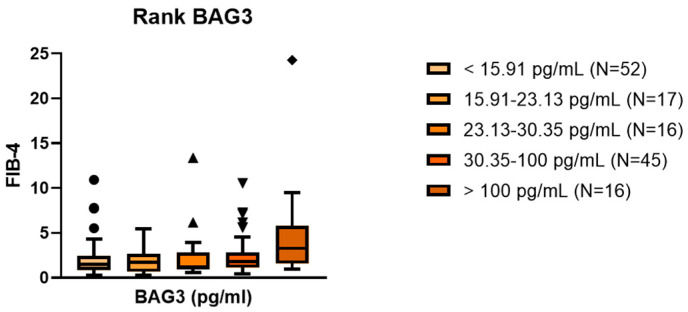
Stepwise association between BAG3 categories and FIB-4. The boxplot illustrates progressive FIB-4 increase across BAG3 categories (<15.91, 15.91–23.13, 23.13–30.35, 30.35–100, >100 pg/mL; *p* = 0.006).

Furthermore, FIB-4 values demonstrated a progressive increase across five BAG3 categories, defined according to healthy control distribution (Kruskal–Wallis *p* = 0.027; Figure 3).

### 2.3. PNPLA3 and TM6F2 Genotypes Influences BAG3 Sera Levels

**Figure 4:**
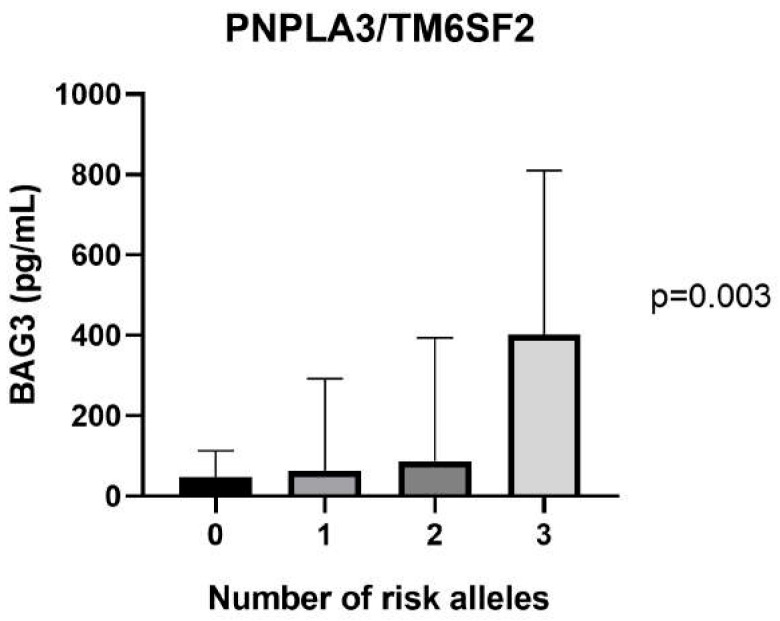
Serum BAG3 levels according to number of risk alleles. Additive effect of combined PNPLA3 and TM6SF2 risk alleles on BAG3 levels, showing stepwise increases. The bar chart shows mean ± SD of BAG3 concentrations for 0, 1, 2, and ≥3 risk alleles.

While BAG3 levels did not significantly differ by *PNPLA3* rs738409 genotype (ANOVA *p* = 0.361), a significant association was observed with *TM6SF2* rs58542926: indeed, T-allele carriers had higher BAG3 concentrations (ANOVA *p* = 0.028). This observation suggests a stronger mechanistic link between *TM6SF2*-mediated lipid retention and ER stress, which may trigger BAG3 upregulation. Importantly, BAG3 levels increased incrementally with the combined number of *PNPLA3* and *TM6SF2* risk alleles, indicating a cumulative genetic effect (ANOVA *p* = 0.003; Figure 4).

## 3. Discussion

EASL’s adoption of the MASLD nomenclature and diagnostic framework represents a paradigm shift in the diagnosis of liver disease, with a focus on early identification of fibrotic risk in metabolically predisposed individuals.

The liver is a stress-sensitive organ, and chronic metabolic insults in MASLD—such as lipotoxicity, oxidative stress, and ER dysfunction—are known inducers of BAG3 expression [17,31]. In this study, we demonstrate for the first time that serum BAG3 concentrations are positively associated with hepatic fibrosis severity in patients with MASLD. These findings add to the growing evidence implicating stress response pathways in fibrogenesis and indicate that BAG3 may serve as a potential non-invasive biomarker candidate for fibrosis assessment in MASLD, pending validation in larger cohorts.

In addition, we propose BAG3 as a possible mediator of proteostasis and fibrogenic progression in the disease. Indeed, the observed positive correlation between serum BAG3 levels and fibrosis severity is consistent with previous observations highlighting BAG3’s role in modulating fibroblast survival and extracellular matrix deposition under stress conditions [18,19,20], and suggests that BAG3 up-regulation might be a mechanistically relevant component of the fibrogenic process.

The progressive increase in BAG3 concentrations across fibrosis stages and genetic risk burden suggests its induction reflects a stress-adaptive yet pro-fibrotic milieu. TM6SF2-associated lipid retention, and to a lesser extent PNPLA3-driven lipotoxicity, may activate endoplasmic reticulum stress pathways, with BAG3 acting as a downstream effector that enables cellular survival at the cost of promoting hepatic stellate cell activation and matrix deposition. Intriguingly, BAG3 levels were associated with *TM6SF2* but not *PNPLA3* genotype, reinforcing a model where *TM6SF2*-driven hepatic lipotoxicity directly engages ER stress and stress-inducible chaperone systems. TM6SF2’s role in reducing VLDL secretion contributes to hepatocyte lipid accumulation, a recognized trigger of unfolded protein response pathways in MASLD pathogenesis [14,15]. This supports a plausible mechanistic link between TM6SF2-mediated lipid retention and BAG3 induction.

The additive effect of *PNPLA3* and *TM6SF2* risk alleles on BAG3 levels further highlights the intersection of metabolic genetics and stress-response biology. This suggests that BAG3 may integrate upstream genetic insults with cellular stress responses and fibrogenic outputs, functioning both as a biomarker and a potential therapeutic checkpoint. Overall, our results support the hypothesis that BAG3 could act as a stress-induced biomarker likely linking genetic susceptibility and fibrotic remodeling in MASLD, although longitudinal studies are necessary to confirm its prognostic role.

## 4. Materials and Methods

### 4.1. Study Population

This cross-sectional study included 146 adults with a clinical diagnosis of metabolic dysfunction-associated steatotic liver disease (MASLD), enrolled at a tertiary care center. All participants self-reported Southern European ancestry.

Inclusion criteria were:

- **Evidence of hepatic steatosis** on imaging (ultrasound or controlled attenuation parameter [CAP]) or liver histology;
- **≥1 cardiometabolic risk factor**, including overweight/obesity, dyslipidaemia, dysglycaemia, or hypertension.

Participants were phenotyped into three groups:

- **Uncomplicated MASLD** (*n* = 121);
- **Cirrhotic MASLD** (*n* = 23);
- **MASLD-related hepatocellular carcinoma (HCC)** (*n* = 2).

The study was conducted according to standard clinical practice protocols and was approved by the local Ethics Committee (CEI Campania Sud IRB n.8/2018). Informed consent was obtained from all participants prior to enrollment.

### 4.2. Fibrosis Assessment

Liver fibrosis was assessed non-invasively using FIB-4 index, calculated from age, AST, ALT, and platelet count [32]. FIB-4 is the most widely established and available tool [33]. However, its ability to detect fibrosis is limited in the intermediate range (1.3–2.67). Notably, a different lower FIB-4 cut-off of 2.0 applies in individuals over 65 years of age.

Risk categories were defined as:

- **Low risk**: <1.30 (or <2.00 if age > 65 years).
- **Intermediate risk**: 1.30–2.67.
- **High risk**: >2.67.

### 4.3. Serum BAG3 Quantification

BAG3 (Bcl-2-associated athanogene 3) concentrations were measured in serum samples using a validated in-house ELISA, performed in duplicate. Methodological details were previously published [30].

Serum BAG3 levels in healthy controls were obtained from a previously published study [30]. Data were used solely as a reference range for BAG3 values. Mean serum BAG3 concentration in healthy subjects was 23.13 ± 10.4 pg/mL, with a 95th percentile of 30.35 pg/mL.

### 4.4. Genotyping

DNA was isolated from peripheral blood using QiAmp^®^ DNA Mini kit (Qiagen, Hilden, Germany), in accordance with the user manual protocol. Quality and quantity were determined with the spectrophotometer Implen NP80 Touch (Implen, München, Germany).

The following SNPs were genotyped using TaqMan^®^ 5′-nuclease SNP assays (ThermoFisher, Waltham, MA, USA):

- *PNPLA3* rs738409 (C>G, p.I148M).
- *TM6SF2* rs58542926 (C>T, p.E167K).

Genotype frequencies were in Hardy–Weinberg equilibrium (χ^2^ *p* > 0.05).

### 4.5. Statistical Analysis

Continuous variables are reported as mean ± standard deviation (SD); categorical data as *n* (%).

Comparisons between groups were performed using ANOVA or Kruskal–Wallis tests.

Spearman’s correlation was used for association testing between BAG3 and FIB-4.

A *p*-value < 0.05 was considered significant. All analyses were conducted using IBM SPSS Statistics v26.

## 5. Limitations and Future Directions

This study’s cross-sectional nature precludes assessment of causal relationships. Liver fibrosis was assessed using the FIB-4 index rather than histopathology, although FIB-4 is well-validated in clinical settings [32]. Additionally, the cohort’s relatively small size and ethnic homogeneity (all participants were from Southern European ancestry) may limit generalizability. While we included two individuals with HCC to illustrate trends at the highest fibrosis stages, the small number prevents any definitive conclusions regarding HCC-specific associations. Future studies should examine longitudinal trajectories of BAG3 expression and assess its utility in predicting fibrosis progression or treatment response. Additionally, mechanistic studies should assess whether BAG3 knockdown or pharmacological modulation alters HSC activation or hepatocyte stress tolerance under lipotoxic conditions, especially in genetic models of MASLD. Targeting BAG3, either to enhance its adaptive roles or block maladaptive signaling, may offer new therapeutic strategies in MASLD.

## 6. Conclusions

The presence and severity of hepatic steatosis and fibrosis should be assessed in all individuals with at least one cardiometabolic risk factor using validated non-invasive blood-based tests, despite their limited accuracy in distinguishing intermediate-risk categories. Most importantly, they do not provide information on the degree of hepatic inflammation. Determining fibrosis risk is essential to identify individuals at an increased risk of developing liver-related complications during early follow-up [34].

This study identifies BAG3 as potentially informative biomarker of hepatic fibrosis in patients with MASLD. Serum BAG3 levels not only correlated with non-invasive fibrosis scores but also increased with the burden of *TM6SF2* and *PNPLA3* risk alleles—highlighting their relevance at the intersection of genetic susceptibility and stress-responsive fibrogenic pathways. These findings suggest that BAG3 implication in MASLD may reflect a previously underappreciated mechanism of fibrotic remodeling linked to hepatocellular stress and metabolic dysfunction.

Importantly, the integration of BAG3 measurements with established genetic and clinical parameters could improve risk stratification and disease monitoring in MASLD. Further longitudinal and mechanistic studies are needed to validate BAG3 as a prognostic biomarker and to clarify whether it might represent a therapeutic target in liver fibrosis.

## Figures and Tables

**Table 1:** Characteristics of study cohort.

Variable	Total (*n* = 146)	MASLD (*n* = 121)	Cirrhosis + HCC (*n* = 25)	*p*-Value
Age (years)	62.9 ± 14.0	61.8 ± 14.8	67.9 ± 8.4	0.020
Male sex	82 (56.2%)	64 (52.9%)	18 (72.0%)	—
BMI (kg/m^2^)	32.3 ± 6.8	32.0 ± 6.8	34.2 ± 6.5	—
AST (U/L)	35.5 ± 23.5	35.6 ± 21.1	**44.7 ± 31.6**	0.040
ALT (U/L)	40.8 ± 33.0	42.9 ± 35.0	30.3 ± 18.1	—
LS (kPa)	9.36 ± 9.3	7.24 ± 4.02	**19.2 ± 17.5**	2.98 × 10^−8^
FIB-4	2.51 ± 2.89	1.74 ± 1.35	**6.25 ± 4.89**	2.11 × 10^−10^
BAG3 (pg/mL)	75.9 ± 226.5	61.8 ± 220.9	**143.8 ± 245.1**	0.011

Abbreviations: BMI: Body Mass Index; AST: Aspartate Aminotransferase; ALT: Alanine Aminotransferase; LS: Liver Stiffness; FIB-4: Fibrosis-4 Index; BAG3: BCL2-Associated Athanogene 3. **Bold** = significantly higher in that group.

## Data Availability

The raw data supporting the conclusions of this article will be made available by the authors on request.

## Supplementary Materials

The following supporting information can be downloaded at: https://www.mdpi.com/article/10.3390/ijms262311286/s1.

## Abbreviations

The following abbreviations are used in this manuscript:

ALT	Alanine Aminotransferase
AST	Aspartate Aminotransferase
BAG3	Bcl-2-associated Athanogene 3
BMI	Body Mass Index
CAP	Controlled Attenuation Parameter
CEI	Comitato Etico Interaziendale (Ethics Committee)
EASD	European Association for the Study of Diabetes
EASL	European Association for the Study of the Liver
EASO	European Association for the Study of Obesity
ELISA	Enzyme-Linked Immunosorbent Assay
ER	Endoplasmic Reticulum
FIB-4	Fibrosis-4 Index
HCC	Hepatocellular Carcinoma
HSC	Hepatic Stellate Cell
Hsp70	Heat Shock Protein 70
IRB	Institutional Review Board
LS	Liver Stiffness
MASLD	Metabolic Dysfunction-Associated Steatotic Liver Disease
PNPLA3	Patatin-like Phospholipase Domain-containing Protein 3
PRS	Polygenic Risk Score
ROS	Reactive Oxygen Species
SNP	Single Nucleotide Polymorphism
TM6SF2	Transmembrane 6 Superfamily Member 2
VLDL	Very-Low-Density Lipoprotein
