# Supplementary material for: Unraveling the Role of BAG3 in Hepatic Fibrosis: Genetic and Biomarker Insights in Metabolic Dysfunction-Associated Steatotic Liver Disease (MASLD)
Source: Int J Mol Sci. 2025 Nov 22;26(23):11286. doi: 10.3390/ijms262311286 (PMC12692393; doi:10.3390/ijms262311286)
Supplement: Supplementary file 1 [file ijms-26-11286-s001.zip › ijms-3972222-supplementary.pdf]

Supplementary Table S1. Detailed clinical and biochemical characteristics of the study cohort.

Variable	Total (n = 146)	MASLD (n = 121)	Cirrhosis (n = 23)	HCC (N=2)	p-value
Age (years)	62.9 ± 14.0	61.8 ± 14.8	**68.4 ± 7.4**	62.0 ± 19.8	0.0496
Male sex	82 (56.2%)	64 (52.9%)	17 (73.9%)	1 (50%)	—
BMI (kg/m^2)	32.3 ± 6.8	32.0 ± 6.8	34.0 ± 6.6	37.6*	—
AST (U/L)	35.5 ± 23.5	33.6 ± 21.1	45.1 ± 33.0	40.0 ± 2.8	—
ALT (U/L)	40.8 ± 33.0	42.9 ± 35.0	31.1 ± 18.7	21.0 ± 0.0	—
LS (kPa)	9.36 ± 9.3	7.24 ± 4.02	**18.5 ± 17.7**	33.0*	1.81×10^{-7}
FIB-4	2.51 ± 2.89	1.74 ± 1.35	**6.26 ± 5.08**	**6.18 ± 2.30**	1.58×10^{-9}
BAG3 (pg/mL)	75.9 ± 226.5	61.8 ± 220.9	**154.2 ± 253.3**	25.0 ± 19.5	0.031

Abbreviations: BMI: Body Mass Index; AST: Aspartate Aminotransferase; ALT: Alanine Aminotransferase; LS: Liver Stiffness; FIB-4: Fibrosis-4 Index; BAG3: BCL2-Associated Athanogene 3. **Bold** = significantly higher in that group.
